# Dosimetric impact of intermediate dose calculation for optimization convergence error

**DOI:** 10.18632/oncotarget.7743

**Published:** 2016-02-26

**Authors:** Byung Do Park, Tae Gyu Kim, Jong Eon Kim

**Affiliations:** ^1^ Department of Radiation Oncology, Samsung Changwon Hospital, Sungkyunkwan University School of Medicine, Changwon, Korea; ^2^ Department of Radiological Science, Kaya University, Gimhae, Korea

**Keywords:** intensity modulated radiation therapy (IMRT), intermediate dose calculation, anisotropic analytical algorithm (AAA)

## Abstract

Intensity-modulated radiation therapy (IMRT) provides the protection of the normal organs and a precise treatment plan through its optimization process. However, the final dose-volume histogram (DVH) obtained by this technique differs from the optimal DVH, owing to optimization convergence errors. Herein, intermediate dose calculation was applied to IMRT plans during the optimization process to solve these issues.

Homogeneous and heterogeneous targets were delineated on a virtual phantom, and the final DVH for the target volume was assessed on the target coverage. The IMRT plans of 30 patients were established to evaluate the usefulness of intermediate dose calculation.

The target coverage results were analogous in the three plans with homogeneous targets. Conversely, conformity indices (conformity index [CI], heterogeneity index [HI], and uniformity index [UI]) of plans with intermediate dose calculation were estimated to be more homogenous than plans without this option for heterogeneous targets (CI, 0.371 vs. 1.000; HI, 0.104 vs. 0.036; UI, 1.099 vs. 1.031 for Phantom B; and CI, 0.318 vs. 0.956; HI, 0.167 vs. 0.076; UI, 1.165 vs. 1.057 for Phantom C). In brain and prostate cancers, a slight difference between plans calculated with anisotropic analytical algorithm (AAA) was observed (HI, *p* = 0.043, UI, *p* = 0.043 for brain; HI, *p* = 0.042, UI, *p* = 0.043 for prostate). All target coverage indices were improved by intermediate dose calculation in lung cancer cases (*p* = 0.043).

In conclusion, intermediate dose calculation in IMRT plans improves the target coverage in the target volume around heterogeneous materials. Moreover, the optimization time can be reduced.

## INTRODUCTION

The development of intensity-modulated radiation therapy (IMRT) has led to significant improvements in dose conformity; however, this technique increases the time needed for the dose calculation and plan optimization. On the contrary, volumetric modulated arc therapy (VMAT) technique developed by Otto reduces the beam delivery time and inter-fractional error by using fewer monitor units than IMRT [1], moreover, VMAT technique improves the target coverage and reduces systematic error in normal organ [2]. Recent progress made in treatment planning technology has led to improvements in the treatment efficiency, as well as to reduced side effects. However, in the radiation treatment planning process, the final dose-volume histogram (DVH) acquired by IMRT or VMAT differs from the optimal DVH obtained via the optimization process. This is caused by inherent errors in the dose calculation algorithm, such as dose calculation for lateral scatter or buildup region errors due to modeling modulator transmission. This issue arises from optimization convergence error, especially in cases of tissue heterogeneity [3, 4].

In recent years, several studies have been conducted on the optimization of convergence error. In one previous study, an improved final DVH was achieved by using the base dose that was acquired from the initial plan to solve the optimization convergence error [5]. Meanwhile, innovative method, named intermediate dose calculation, has been previously applied to the VMAT technique with the jaw tracking option in Progressive resolution optimizer (PRO3) algorithm [6], it was applied to IMRT plans in Eclipse version 11 (Varian Medical Systems, Palo Alto, CA) for the first time in 2009 by Zacarias and Mills [4].

Intermediate dose calculation is carried out during the optimization of the initial plan, after which optimization is continuously performed with the calculated dose distribution; subsequently, the dose calculation algorithm is applied to the treatment plan once more after the optimization is finished. Intermediate dose calculation is essential to ensure an improved and rapid optimization process and target coverage. In addition, for accurately calculating the optimization for heterogeneous materials, Acuros XB (AXB), a specific dose calculation algorithm, was developed [7]. AXB algorithm is similar to classic Monte Carlo method for calculating of dose distribution in heterogeneous material, on the other hand, anisotropic analytical algorithm (AAA) is the dose calculation algorithm base on a pencil beam convolution-superposition method [8]. AAA algorithm in dose calculation improved the weakness of pencil beam convolution algorithm in heterogeneous material. However this algorithm still has the dosimetric error in soft tissue. Whereas, several studies reported that AXB is superior to AAA in inhomogeneous and heterogeneous material [9–11].

This study aimed to investigate the dosimetric impact of intermediate dose calculation by DVHs, and we moreover briefly examine the dosimetric differences between AAA and AXB algorithms.

## RESULTS

### Phantom study

All results derived from the phantom study are summarized in Table 1. The target coverages investigated with Phantom A showed similar results, and the maximum doses for the target volumes were 102.6%, 101.8%, and 102.6% in Plan1, Plan1-DC, and Plan1-int, respectively. On the other hand, the maximum doses were increased in Phantoms B and C, which included heterogeneous materials. The maximum doses in Phantom B was 99.7%, 101.6%, and 101.8%, respectively, in Plan1, Plan1-DC, and Plan1-int, while the maximum doses in Phantom C were 99.2%, 102.8%, and 103.9%, respectively. As a result, we achieved measurement results with non-negligible differences for the target coverage compared to those in Phantom A.

**Table 1:** Comparisons of the target coverage in each phantom

		Plan1	Plan1-DC	Plan1-int
Phantom A	CI	1.000	1.000	1.000
	HI	0.030	0.026	0.032
	UI	1.026	1.023	1.028
Phantom B	CI	0.371	1.000	1.000
	HI	0.104	0.039	0.036
	UI	1.099	1.034	1.031
Phantom C	CI	0.318	0.930	0.956
	HI	0.167	0.070	0.076
	UI	1.165	1.051	1.057

CI: conformity index, HI: heterogeneity index, UI: uniformity index.

**Figure 1:**
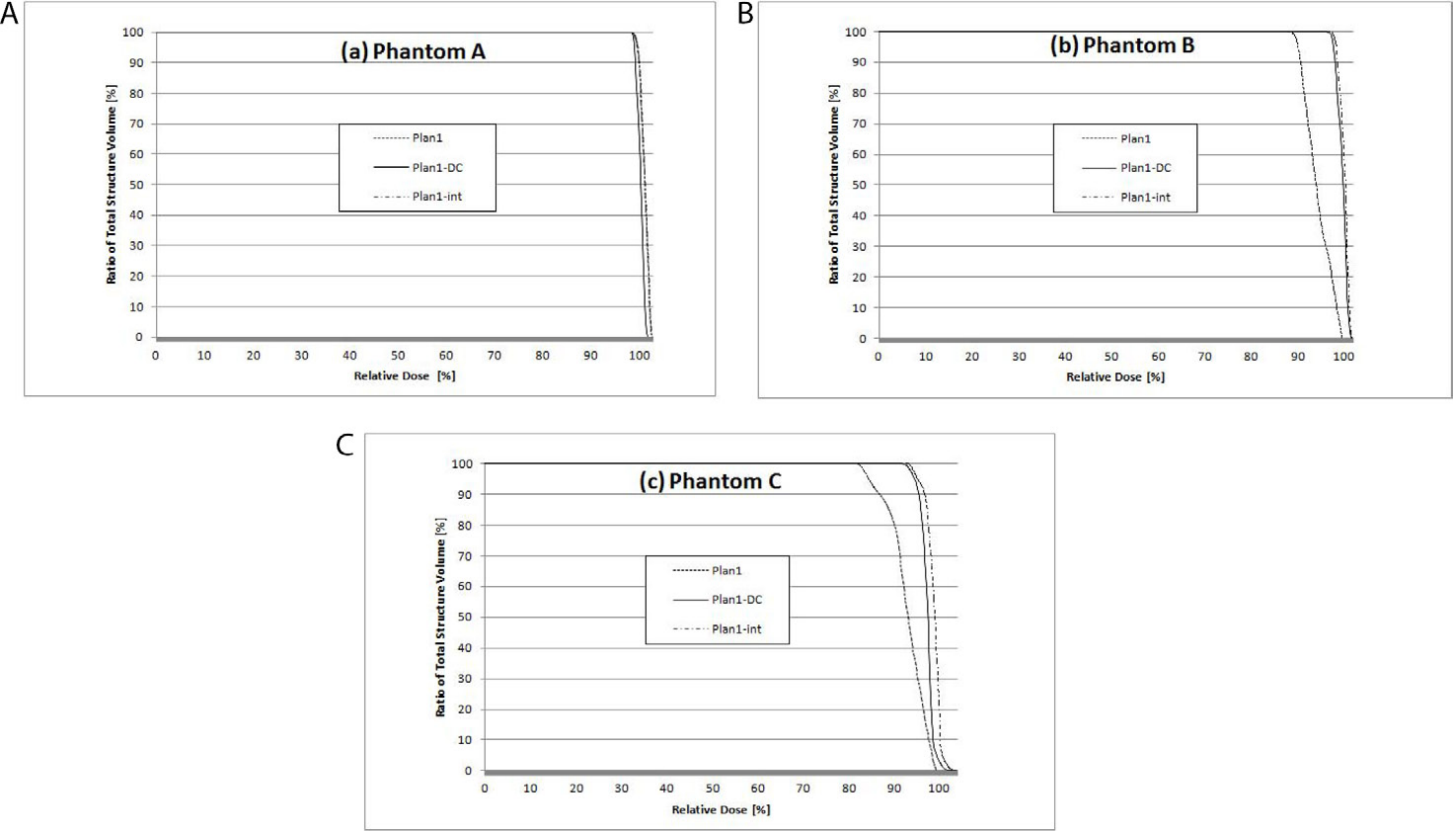
Dose volume histograms of the target volume in each phantom (**a**) Phantom A, (**b**) Phantom B, and (**c**) Phantom C. Dotted line = plan1; solid line = plan1-DC; dot-and-dashed line = plan1-int.

DVH results of the target volume are plotted in Figure 1. DVHs of all plans in Phantom A (Figure 1. (a)) showed similar results, whereas DVH results for the target volume in Plan1-DC and Plan1-int were significantly improved by using intermediate dose calculation (Figure 1. (b) and (c)).

### Clinical cases

**Figure 2:**
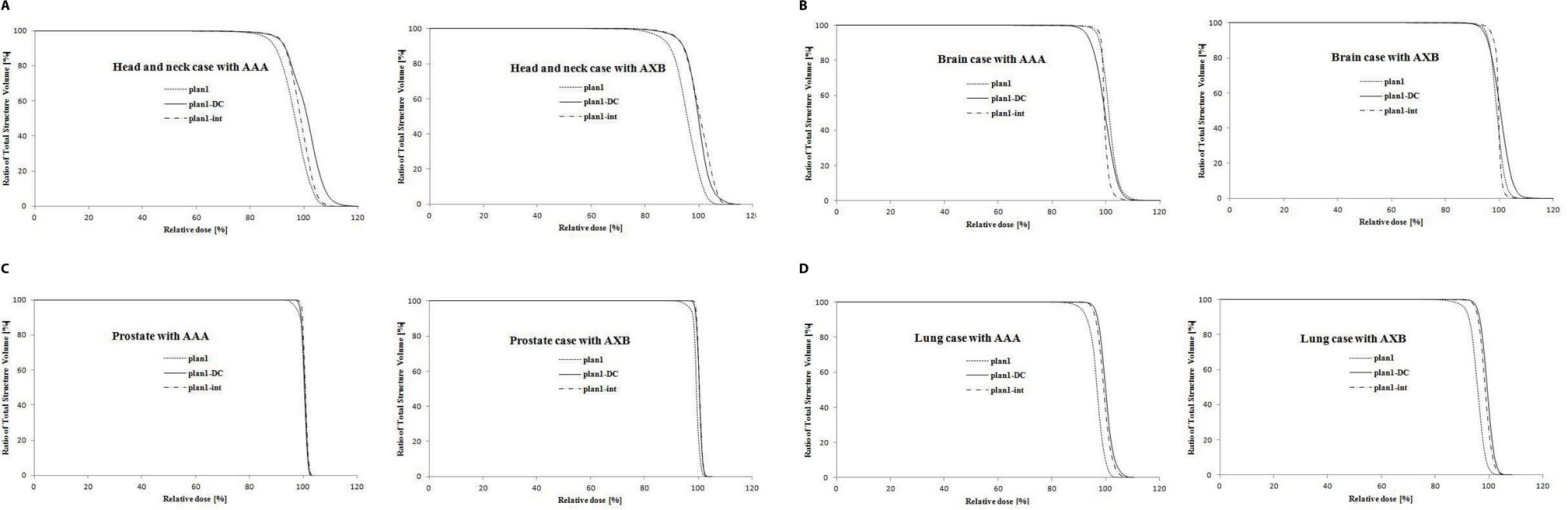
Dose-volume histograms of the target volumes calculated with anisotropic analytic algorithm (AAA) and Acuros XB (AXB) algorithm in Plan1, Plan1-DC, and Plan1-int for head and neck, brain, prostate, and lung cancer cases Dotted line = plan1; solid line = plan1-DC; dot-and-dashed line = plan1-int.

Figure 2 shows DVHs of example cases in each treatment region. The dose distributions were calculated by AAA and AXB algorithms. The target coverage was analyzed by DVHs for 30 cancer cases and evaluated by the mean value and standard deviation of CI, HI and UI which are measured on treatment planning system.

All results calculated by AAA are summarized in Table 2. As a result, in the case of HN, no clinical difference was found. On the other hand, significant clinical differences were observed for the HI (*p* = 0.043) and UI (*p* = 0.043) between Plan1 and Plan1-int in brain cancer cases, and for HI (*p* = 0.042) between Plan1 and Plan1-DC and UI (*p* = 0.043) between Plan1 and Plan1-int in prostate cancer cases. Table 3 shows the results obtained by AXB. This result showed a similar tendency to those obtained by AAA. In the case of lung cancer, considerable differences in both AAA and AXB algorithms were observed for the target coverage upon intermediate dose calculation, indicating that DVH can be achieved accurately by intermediate dose calculation for the treatment target around heterogeneous material.

**Table 2:** Summary of each parameter measured by AAA for each region

AAA		Plan1	Plan1-DC	Plan1-int	*p*-value	
					Plan1 vs. Plan1-DC	Plan1 vs. Plan1-int
Head and neck						
CI	Mean	0.833	0.913	0.833	0.221	0.925
	SD	0.221	0.084	0.190		
HI	Mean	0.126	0.139	0.108	0.320	0.073
	SD	0.086	0.090	0.085		
UI	Mean	1.099	1.117	1.083	0.132	0.147
	SD	0.060	0.073	0.061		
Brain						
CI	Mean	0.981	0.967	0.973	0.715	0.273
	SD	0.017	0.052	0.030		
HI	Mean	0.094	0.096	0.077	0.892	0.043
	SD	0.031	0.049	0.023		
UI	Mean	1.072	1.077	1.058	0.893	0.043
	SD	0.023	0.042	0.019		
Prostate						
CI	Mean	0.974	1.000	1.000	0.068	0.068
	SD	0.032	0.000	0.000		
HI	Mean	0.066	0.043	0.031	0.042	0.043
	SD	0.016	0.005	0.010		
UI	Mean	1.053	1.036	1.024	0.043	0.042
	SD	0.016	0.005	0.008		
Lung						
CI	Mean	0.647	0.982	0.961	0.043	0.043
	SD	0.305	0.023	0.036		
HI	Mean	0.161	0.088	0.085	0.043	0.043
	SD	0.043	0.023	0.022		
UI	Mean	1.140	1.070	1.069	0.043	0.043
	SD	0.052	0.019	0.019		

AAA: anisotropic analytical algorithm, CI: conformity index, HI: heterogeneity index, UI: uniformity index, SD: standard deviation.

**Table 3:** Summary of each parameter measured by AXB algorithm for each region

AXB		Plan1	Plan1-DC	Plan1-int	*p*-value	
					Plan1 vs. Plan1-DC	Plan1 vs. Plan1-int
Head and neck						
CI	Mean	0.778	0.923	0.779	0.010	0.826
	SD	0.256	0.071	0.242		
HI	Mean	0.125	0.137	0.118	0.443	0.426
	SD	0.075	0.084	0.087		
UI	Mean	1.100	1.109	1.095	0.510	0.495
	SD	0.060	0.063	0.067		
Brain						
CI	Mean	0.940	0.978	0.959	0.138	0.138
	SD	0.058	0.033	0.040		
HI	Mean	0.092	0.095	0.084	0.715	0.465
	SD	0.025	0.040	0.030		
UI	Mean	1.072	1.076	1.063	0.892	0.285
	SD	0.021	0.035	0.024		
Prostate						
CI	Mean	0.975	1.000	0.999	0.043	0.043
	SD	0.026	0.000	0.001		
HI	Mean	0.068	0.044	0.034	0.043	0.043
	SD	0.011	0.004	0.008		
UI	Mean	1.051	1.035	1.027	0.042	0.043
	SD	0.010	0.004	0.006		
Lung						
CI	Mean	0.546	0.952	0.953	0.043	0.043
	SD	0.294	0.062	0.049		
HI	Mean	0.172	0.093	0.089	0.043	0.043
	SD	0.059	0.028	0.026		
UI	Mean	1.144	1.077	1.072	0.043	0.043
	SD	0.067	0.025	0.021		

AXB: Acuros XB, CI: conformity index, HI: heterogeneity index, UI: uniformity index, SD: standard deviation.

**Figure 3:**
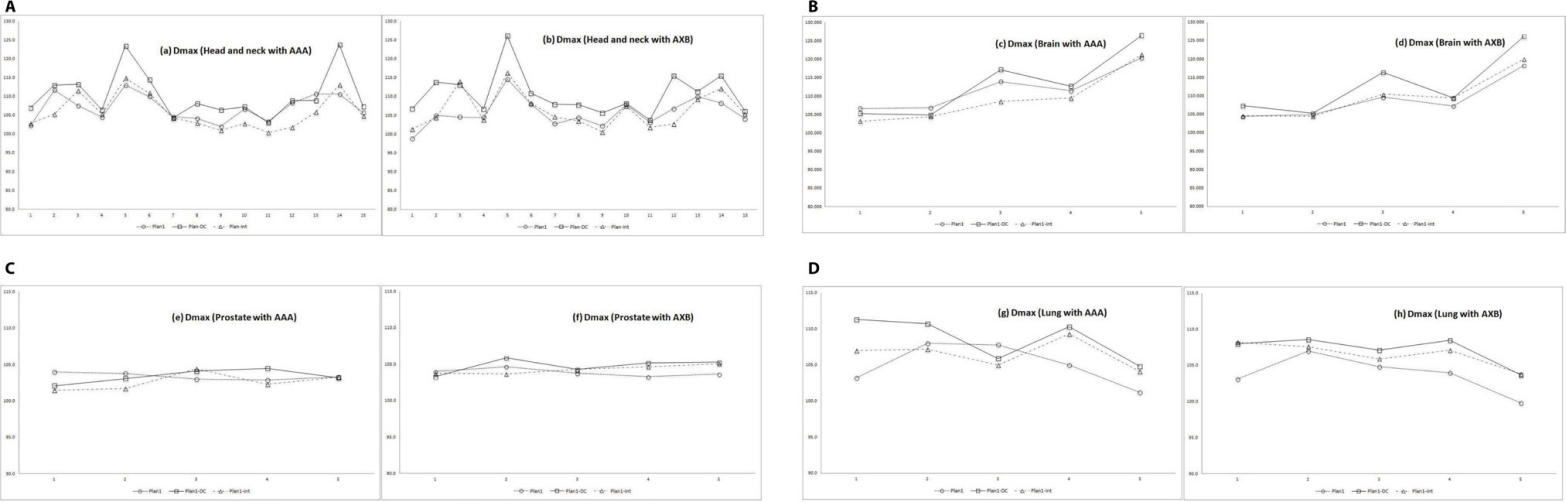
Maximum dose distributions for the target volumes in each region Dotted line = plan1; solid line = plan1-DC; dot-and-dashed line = plan1-int. The y-axis shows the dose maximum value (Dmax).

The dose maximum values by AAA and AXB algorithms for the target volume are plotted in Figure 3. In Plan1-DC, the average maximum values by AAA were 110.4%, 113.3%, 103.4%, and 108.6% in HN, brain, prostate, and lung cancer cases, respectively. The corresponding average maximum values by AXB were 110.7%, 113.0%, 104.8%, and 107.2%, respectively. These results were higher than those for Plan1 and Plan1-int.

**Figure 4:**
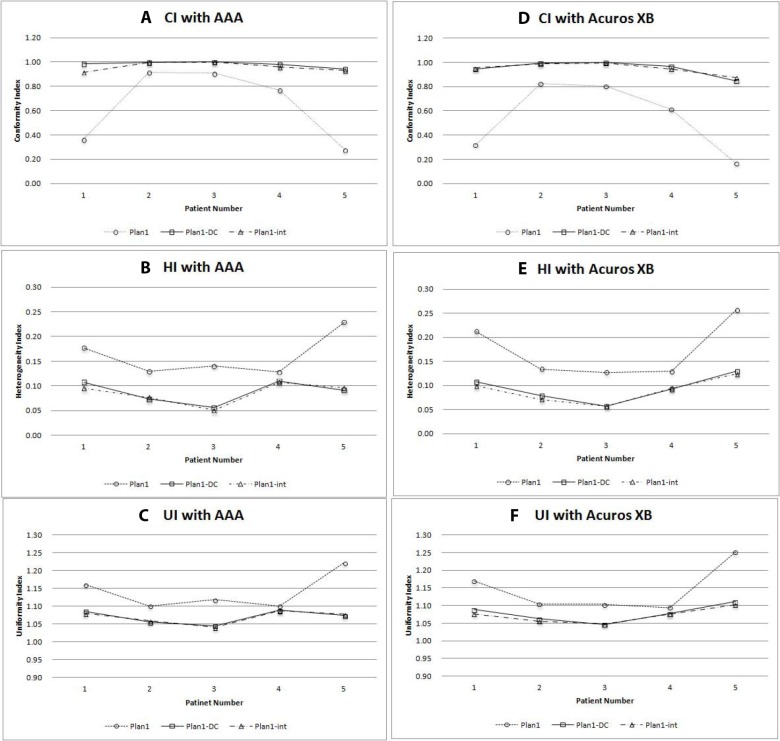
Comparison of conformity index (CI), heterogeneity index (HI), and uniformity index (UI) in each plan calculated by anisotropic analytic algorithm (AAA) (A, B, C), and Acuros XB (AXB) algorithm (D, E, F) for 5 lung cancer cases Dotted line = plan1; solid line = plan1-DC; dot-and-dashed line = plan1-int.

Figure 4 shows the target coverage calculated by AAA and AXB algorithms in lung cancer cases. The result revealed that dose distribution by using intermediate dose calculation showed better target coverage in both AAA and AXB algorithms. A comparative study of the dosimetric impact on AAA and AXB algorithms for lung cancer cases are illustrated in Table 4.

**Table 4:** AAA vs. AXB for 5 lung cancer cases

	Target volume	Relative difference (%)	*p*-value	Lung volume	Relative difference (%)	*p*-value
Plan1	D98%	−2.73	0.043	V5Gy	1.16	0.500
	D2%	−1.44	0.043	V20Gy	−1.64	0.138
	Dmean	−1.21	0.042	MLD	−0.47	0.357
Plan1-DC	D98%	−1.61	0.080	V5Gy	2.78	0.043
	D2%	−1.11	0.080	V20Gy	−0.12	0.893
	Dmean	−0.88	0.080	MLD	0.53	0.257
Plan1-int	D98%	−1.04	0.080	V5Gy	2.33	0.225
	D2%	−0.60	0.138	V20Gy	−0.33	0.686
	Dmean	−0.26	0.343	MLD	−1.24	0.074

AAA: anisotropic analytical algorithm, AXB: Acuros XB, D_2/98%_: average dose covering 2% or 98% of the target volume, V_5/20Gy_: average lung volume receiving 5 Gy or 20 Gy, MLD: mean lung dose. Relative difference: (AXB − AAA)/AAA × 100.

A minor relative difference was observed between AAA and AXB algorithms, indicating that the target coverage was lower in AXB plans than in AAA. In Plan1, significant differences between AAA and AXB algorithms for D_98%_, D_2%_, and D_mean_ were observed, whereas these significant differences were reduced between Plan1-DC and Plan1-int. These results were similar to the values observed in the other regions (Table 5).

**Table 5:** AAA vs. AXB for head and neck, brain, and prostate cancer cases (Relative difference)

	Target volume	HN	Brain	Prostate
		Relative difference (%)	Relative difference (%)	Relative difference (%)
Plan1	D_98%_	−1.34	−2.48	−0.60
	D_2%_	−1.67	−2.59	−0.41
	D_mean_	−1.53	−1.77	−0.74
Plan1-DC	D_98%_	−0.96	0.47	−0.27
	D_2%_	−0.87	0.37	−0.21
	D_mean_	−0.05	0.26	−0.24
Plan1-int	D_98%_	−0.60	−1.18	−0.28
	D_2%_	−0.03	−0.37	0.05
	D_mean_	−0.40	−0.22	−0.18

AAA: anisotropic analytical algorithm, AXB: Acuros XB algorithm, D_2/98%_: average dose covering 2% or 98% of the target volume, V_5/20Gy_: average lung volume receiving 5 Gy or 20 Gy, MLD: mean lung dose, HN: head and neck. Relative difference = (AXB − AAA)/AAA × 100.

## DISCUSSION

Treatment plans using IMRT and VMAT techniques are associated with a precise target volume and minimized side effects due to enhanced protection of the normal organ. However, many trials are currently required to achieve an effective treatment plan during the optimization process, and this is associated with a high rate of error.

This study assessed the dosimetric impact of intermediate dose calculation applied to IMRT technique using Eclipse version 11. Our study revealed that intermediate dose calculation is a useful function for IMRT plans. Especially, the target coverage was improved for target volumes using the intermediated calculation method.

In the study by Zacarias and Mills, the final dose distribution was found to be consistent with the result of the repeated optimization process based on the original plan, and the authors therefore concluded that the intermediate dose option is reasonable [4]. Further, Kan et al. reported that there was no difference according to the application of intermediate dose calculation in their phantom study. However, the authors used VMAT technique and evaluated the results according to application of an air cavity correction option simultaneously with the intermediate dose option [20]. Li et al. presented the effect of the intermediate dose calculation for lung cancer cases. They reported that dose homogeneity and optimization efficiency were improved by utilizing the intermediate dose calculation [21].

Various dose calculation algorithm have been developed to ensure accurate dose calculation. Fogliata et al. reported that AXB algorithm can be an alternative to the Monte Carlo calculation [9], and Kan et al. demonstrated that AAA may result in overestimations in small fields [22]. Further, it has been reported that in treatment plans with IMRT or VMAT technique, there is a difference between AAA and AXB algorithms [23].

In the present study, we evaluated the adequacy of intermediate dose calculation for four kinds of treatment regions using IMRT technique. Despite the limited number of clinical cases, this function was found to be effective in obtaining an accurate final DVH. Finally, this study also proposed which dose calculation algorithm should be considered for obtaining a better dose distribution for heterogeneous media through analysis between AAA and AXB algorithms with intermediate dose calculation.

## CONCLUSIONS

This study analyzed DVHs according to the application of intermediate dose calculation. In conclusion, we found that intermediate dose calculation in IMRT plans improved the target coverage in target volumes surrounded by heterogeneous materials such as target volume in prostate and lung cancer cases. Moreover, the optimization time can be reduced during the optimization process. Therefore, it seems reasonable to conclude that intermediate dose calculation should be applied to IMRT plans. However, the full impact of optimization convergence error remains to be investigated. In the comparison of the differences in dose distribution between AAA and AXB algorithms, our results suggest that the dose calculation should be considered by using the developed AXB algorithm.

## MATERIALS AND METHODS

### Phantom study

**Figure 5:**
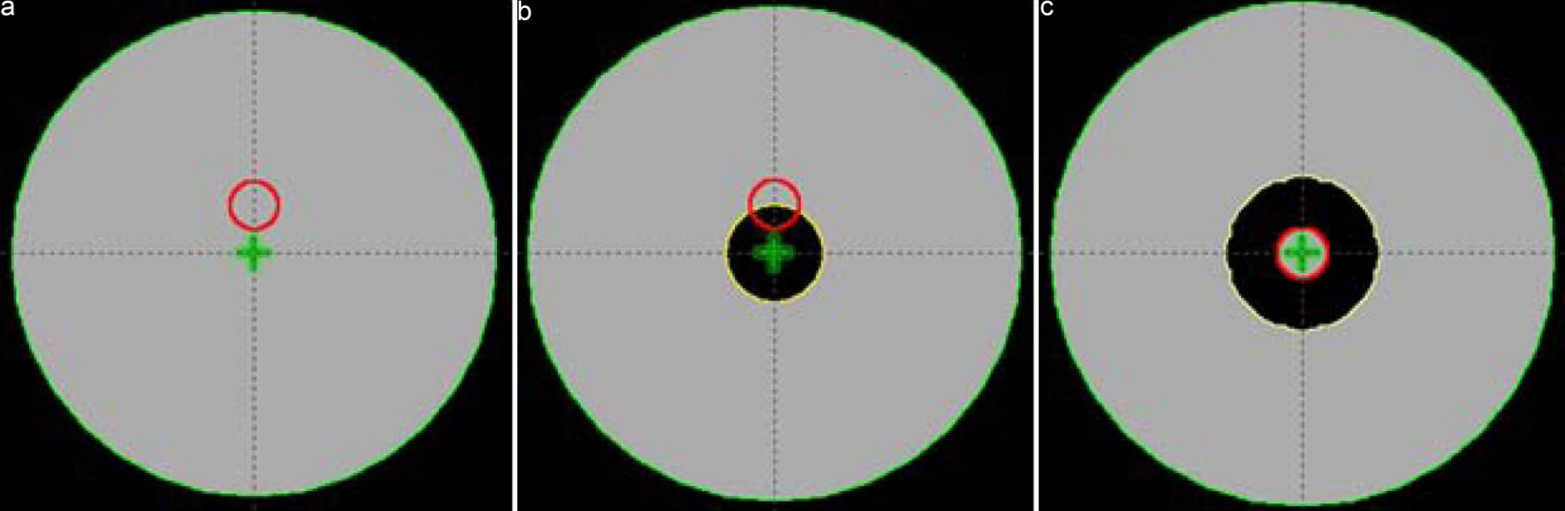
Axial views of the central slice for (a) Phantom A (homogeneous material), (b) Phantom B (small air cavity), and (c) Phantom C (air cavity around the target)

It is necessary to evaluate the dosimetric impact of intermediate dose calculation with a phantom. First, we created three kinds of virtual water phantoms in the treatment planning system: Phantoms A, B, and C. The target volume was created with a diameter of 2 cm in homogeneous Phantom A. An air cavity, with a diameter of 4 cm, was formed in Phantom B, with the diameter of the target volume in Phantom B being the same as that of Phantom A, but including part of the air cavity. In Phantom C, the target volume was delineated with a diameter of 2 cm in the center, and a ring with a width of 2 cm was filled around the target volume (Figure 5). A treatment plan using 6 MV photons with 9 fields was generated for each phantom, and the fields were equally spaced at 40° intervals. IMRT technique was applied to all plans, and dose calculation was performed by AAA.

To analyze the dosimetric impact of the intermediate dose calculation, three kinds of treatment plans were established for each phantom. The first plan was created by optimization and dose calculation without intermediate dose calculation (Plan1). The second plan was generated by re-optimizing and re-calculating Plan1 (Plan1-DC). The dose distribution of Plan1 was used as the intermediate dose at this time. The third plan was produced by optimization and dose calculation with intermediate dose calculation from the beginning (Plan1-int).

The target volume coverage was evaluated by the conformity, heterogeneity, and uniformity, using DVHs in Eclipse. Conformity index (CI), heterogeneity index (HI), and uniformity index (UI) were defined as follows [12]:

$$\text{CI}=\frac{TV_{95\%}}{\text{TV}},$$

where TV is the target volume and TV_95%_ is the volume covered by 95% of the prescribed dose;

$$\text{HI}=\frac{D_{2\%}-D_{98\%}}{D_{50\%}},$$

where D_2%_ is the dose of 2% of the target volume, D_98%_ is the dose of 98% of the target volume, and D_50%_ is the dose of 50% of the target volume [13]; and

$$\text{UI}=\frac{D_{5\%}}{D_{95\%}},$$

where D_5%_ is the dose of 5% of the target volume, and D_95%_ is the dose of 95% of the target volume [14].

### Clinical cases

In head and neck (HN) and brain cancer cases, the treatment target volume is complex, and critical normal organs such as the parotid grand, optic apparatus, and spinal cord are adjacent to the target volume. Therefore, a sharp dose response curve and precise dose calculation are required. In a previous study, IMRT technique showed excellent results for these cases in comparison with three-dimensional conformal radiation therapy [15, 16]. In addition, IMRT technique has also been applied to the treatment plan in order to improve the treatment toxicity and the disease-free survival in prostate cancer cases [17, 18], while, in lung cancer cases, side effects such as pneumonia can be reduced by protecting the healthy lung, esophagus, heart, and spinal cord using this technique [19]. Thus, using IMRT technique in the treatment plan can also improve the dose escalation for these treatment regions.

In this study, CT scans of 30 patients were randomly chosen to assess the dosimetric impact of intermediate dose calculation. The treatment regions of these patients were in the HN (*n* = 15), brain (*n* = 5), prostate (*n* = 5), and lung (*n* = 5). The images were obtained with 2.5 mm slice thickness from 3D-computed tomography (GE Health-care, Buckinghamshire, UK). Three kinds of treatment plans were generated for each image, similar to in the phantom study. The same constraints and priorities in the optimization process were applied to the set of plans (Plan1, Plan1-DC, and Plan1-int). Dose calculation of these treatment plans was carried out by AAA and AXB algorithms.

To evaluate the dosimetric impact of intermediate dose calculation, the target coverage for IMRT plans was estimated by CI, HI, and UI through DVH. The dose distribution by AAA was compared with that by AXB for each treatment region. For the statistical analyses, the Statistical Program for Social Sciences (SPSS version 18; SPSS Inc., Chicago, IL) was used.
